# Supplementary figures and images for: Small-scale drivers on plant and ant diversity in a grassland habitat through a multifaceted approach
Source: PeerJ. 2021 Dec 24;9:e12517. doi: 10.7717/peerj.12517 (PMC8711281; doi:10.7717/peerj.12517)

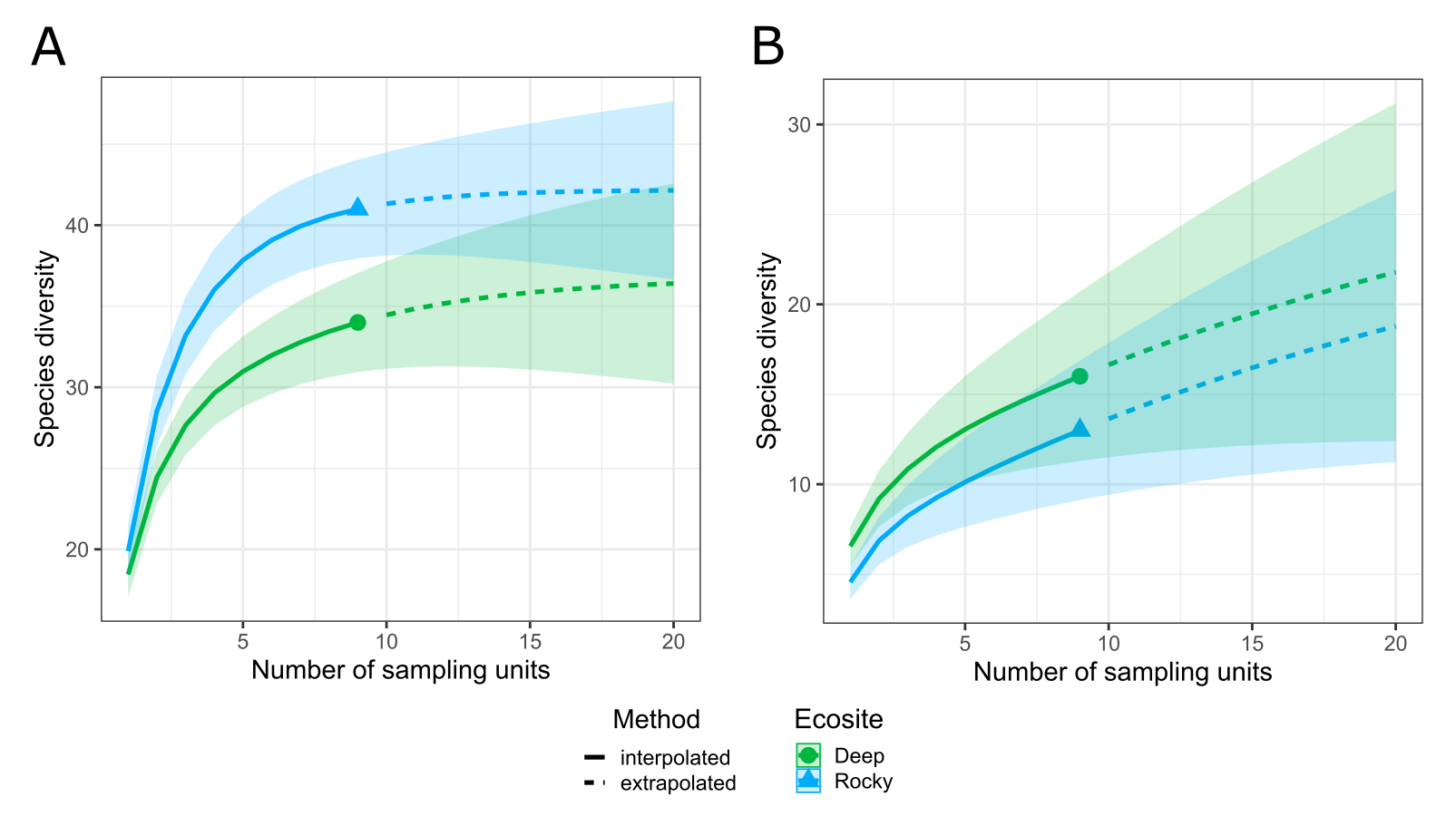

Supplement: Supplemental Information 1 [file peerj-09-12517-s001.png]
